# Environmental transmission from bamboo rats: Mapping current and future talaromycosis risk in China under climate change

**DOI:** 10.1016/j.onehlt.2025.101264

**Published:** 2025-11-01

**Authors:** Nan Xu, Xiaoyun Min, Kunyi Wu, Ting La, Bo Cao

**Affiliations:** a Department of Clinical Laboratory, The Second Affiliated Hospital of Xi'an Jiaotong University, Xi'an 710004, China; b Department of Infectious Diseases, The Second Affiliated Hospital of Xi'an Jiaotong University, Xi'an 710004, China; c Core Research Laboratory, The Second Affiliated Hospital of Xi'an Jiaotong University, Xi'an 710004, China; d National-Local Joint Engineering Research Center of Biodiagnosis & Biotherapy, The Second Affiliated Hospital of Xi'an Jiaotong University, Xi'an 710004, China; e College of Life Sciences, Shaanxi Normal University, Xi'an 710119, China; f Key Laboratory of Surgical Critical Care and Life Support (Xi'an Jiaotong University), Ministry of Education, Xi'an 710004, China

**Keywords:** Talaromycosis, *Talaromyces marneffei*, Bamboo rat, Risk distribution, Maxent

## Abstract

**Background:**

Talaromycosis (TSM), a severe fungal infection caused by *Talaromyces marneffei* (TM), poses a significant threat to immunocompromised individuals in recent years. Despite its high mortality and socioeconomic burden, predictive spatial risk distributions are lacking.

**Methods:**

Here, we first employed a One Health framework to model current and future TM/TSM risk distributions across China. Using the Maximum Entropy (Maxent) model, we predicted the habitat suitability for three key bamboo rat reservoir species. Based on this, we developed the risk distribution maps of TM/TSM across China by integrating socio-economic factors.

**Results:**

Accurate modeling results (AUC: 0.958–0.999) identified precipitation- and temperature- related factors as decisive environmental drivers. Specifically, high-suitability regions were concentrated in Southern/Southwestern China (Yunnan, Guangxi, Guizhou, Sichuan, Hunan, and Guangdong Provinces), coincident with known endemic areas. Furthermore, human infection risk maps were generated by integrating suitability distribution with socio-economic factors (HIV incidence, population density, GDP). High-risk hotspots stratified by HIV status confirmed core endemic zones for HIV-positive populations and identified broader risk areas for HIV-negative populations (e.g., parts of Guangdong, Jiangxi and Fujian Provinces). Projections under different climate change scenarios showed host suitability will decrease under low emissions (SSP126) but expand under high emissions (SSP585), indicating dynamic future TM/TSM risk patterns and disease administration dependent on emission conditions.

**Conclusions:**

Collectively, these findings first revealed high-resolution predictive risk maps of TM/TSM in China and provided valuable reference for targeted public health interventions. The proposed methods in this study will also shed light on the prevention and administration of other “fungi - animal host - human” diseases in both current and emerging risk zones under climate change in the future.

## Graphical abstract

**Figure:**
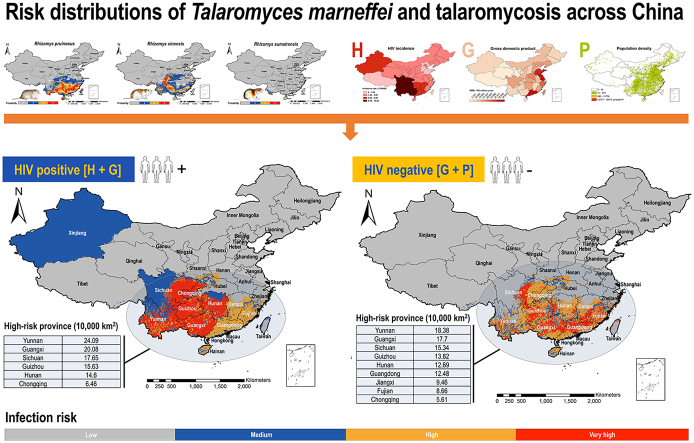

## 1 Introduction

Talaromycosis (TSM), a life-threatening systemic mycosis caused by the dimorphic fungus *Talaromyces marneffei* (TM), represents a serious threat to immunocompromised populations, particularly those with HIV/AIDS, as well as individuals with conditions such as cancer or those on long-term steroid therapy, in Southeast Asia and southern China [1,2]. This neglected tropical disease proliferates at the intersection of environmental reservoirs, zoonotic hosts (specifically bamboo rats), and human susceptibility [3,4]. As the pathogen of TSM, *T. marneffei* exhibits distinctive thermal dimorphism: existing as a saprophytic mold producing infectious conidia in cool soils (< 25 °C) and transitioning to a pathogenic yeast form within human hosts at physiological temperatures (37 °C) [[5], [6], [7]]. This adaptation facilitates disseminated infection. In endemic regions, TSM affects 4–15 % of HIV-positive individuals, frequently signifying advanced AIDS, and carries a case fatality rate exceeding 30 % despite antifungal therapy [8,9]. Clinical manifestations are highly heterogeneous, ranging from non-specific symptoms (e.g., fever, weight loss, anemia) to pathognomonic skin lesions, hepatosplenomegaly, and severe respiratory involvement [10]. Specifically, TSM diagnosis is frequently delayed due to protean early presentations and limited access to advanced microbiological or molecular diagnostics in resource-constrained settings [11]. Beyond its substantial morbidity and mortality, TSM imposes considerable socioeconomic burdens through prolonged hospitalizations, expensive diagnostics and antifungals, income loss, and premature death [12]. Furthermore, it strains healthcare systems concurrently managing high burdens of HIV and tuberculosis. High mortality, diagnostic complexities, and significant resource demands highlight talaromycosis as a persistent yet underprioritized global health challenge requiring enhanced surveillance, preventive strategies, and therapeutic interventions.

The geographic distribution of TSM exhibits significant heterogeneity, concentrated predominantly within the tropical and subtropical zones lying between latitudes 20°N and 20°S [13,14]. This distinct spatial patterning is not random but arises from a complex interplay of ecological and anthropogenic factors acting synergistically. Key environmental determinants include consistently high ambient temperatures (often >25 °C), elevated humidity levels (> 80 %), and specific rainfall patterns associated with monsoon seasons, which collectively favor the survival and proliferation of *T. marneffei* in its environmental niche [15]. In particular, the ecology of specific rodent reservoirs, namely burrow-dwelling bamboo rats, is intrinsically linked to this environmental suitability [16]. Human socio-behavioral factors, particularly agricultural practices, deforestation, and other land-use changes that bring individuals into closer proximity with contaminated environments, further modulate exposure risk [17,18]. Critically, anthropogenic climate change is now dynamically reshaping these fundamental determinants of disease distribution and transmission intensity: rising mean temperatures extend fungal survival and potentially growth rates in soil substrates; altered precipitation regimes and increased frequency of extreme weather events (droughts, floods) modify the structure and health of bamboo forest ecosystems crucial for reservoir hosts; and events like heavy rainfall or soil disturbance may enhance the aerosolization and dispersal of infectious conidia [[19], [20], [21], [22]]. Epidemiological evidence robustly supports this climate-disease link. Previous studies demonstrate strong statistical correlations between seasonal surges in humidity and concurrent increases in talaromycosis hospital admissions [23,24]. Despite these clear signals and the substantial public health impact, high-resolution spatial risk models capable of predicting current and future transmission hotspots at scales relevant for intervention planning remain underdeveloped. This represents a critical gap hindering efficient resource allocation and proactive public health measures targeting this WHO-classified neglected disease.

The bamboo rats of the subfamily Rhizomyinae (particularly *Rhizomys pruinosus*, *R. sinensis*, and *R. sumatrensis*) are unequivocally established as the primary environmental reservoirs of *T. marneffei*, forming an indispensable component of the pathogen's life cycle [2,16]. These rodents harbor the fungus asymptomatically within their reticuloendothelial system (spleen, liver, lymph nodes) at significantly high prevalence rates, ranging from 15 % to 40 % in endemic areas [17]. Specifically, infected rats continuously shed *T. marneffei* conidia into the soil lining their extensive underground burrow systems through excretions and potentially upon death and decomposition [18]. This contamination creates persistent environmental foci of infection. Human acquisition occurs almost exclusively through the inhalation of aerosolized conidia released from this contaminated soil during activities such as farming, digging, construction, foraging for bamboo shoots, or simply living in close proximity to disturbed rat habitats. Consequently, the spatial distribution and habitat suitability for these specific bamboo rat species act as a primary proxy and direct driver for zoonotic exposure risk. Previous studies reveal that high-incidence regions of TSM were highly coincident with suitable habitat of bamboo rats [25,26]. However, these reservoir distributions are not static. They are undergoing significant shifts driven by the dual pressures of climate change (e.g., warming temperatures enabling the northward expansion of suitable habitats in Southern China) and escalating anthropogenic pressures including deforestation, agricultural expansion, urbanization, and potentially wildlife trade [27]. This intricate “host-pathogen-environment” relationship highlights the absolute necessity of understanding the ecological drivers governing reservoir distribution and abundance as a fundamental prerequisite for accurately predicting and mitigating spillover risk to human populations.

Ecological niche modeling (ENM) has emerged as a powerful and indispensable tool for predicting the geographic distribution of species based on their environmental requirements, making it highly applicable to forecasting reservoir habitat suitability and, by extension, zoonotic disease risk [28]. For instance, a recent large-scale serological and modeling study on Oropouche virus (OROV) utilized the ENM method to successfully predict high-risk transmission areas of OROV across Latin America [29]. Among the diverse ENM approaches, the maximum entropy (Maxent) modeling algorithm demonstrates particular strengths for this application [30,31]. Maxent excels at leveraging often limited species occurrence records (presence-only data) in conjunction with a diverse suite of multiscale environmental predictors, including bioclimatic variables (temperature, precipitation patterns), remotely sensed vegetation indices (e.g., normalized difference vegetation index, normalized difference vegetation index [NDVI]), soil characteristics (pH, type), and topographic features [32]. Its foundation in machine learning allows it to effectively model complex, non-linear interactions between these predictors while inherently minimizing the risk of overfitting, a common pitfall with some other methods. Comparative studies consistently show Maxent outperforming traditional regression-based modeling approaches for species distribution prediction, often achieving significantly higher predictive accuracy [33]. The practical utility of Maxent is evidenced by its successful application in predicting distribution shifts for other environmentally mediated zoonoses, such as the emergence of *Cryptococcus gattii* in the Pacific Northwest and the changing risk landscapes for *Rickettsia japonica*, predictions subsequently validated through targeted field surveillance efforts [34,35]. This proven adaptability and robustness make Maxent exceptionally well-suited for the task of quantifying how current habitats of bamboo rats, and their projected shifts under climate change scenarios, directly modulate the spatial and temporal patterns of *T. marneffei* spillover risk to humans.

**Fig. 1:**
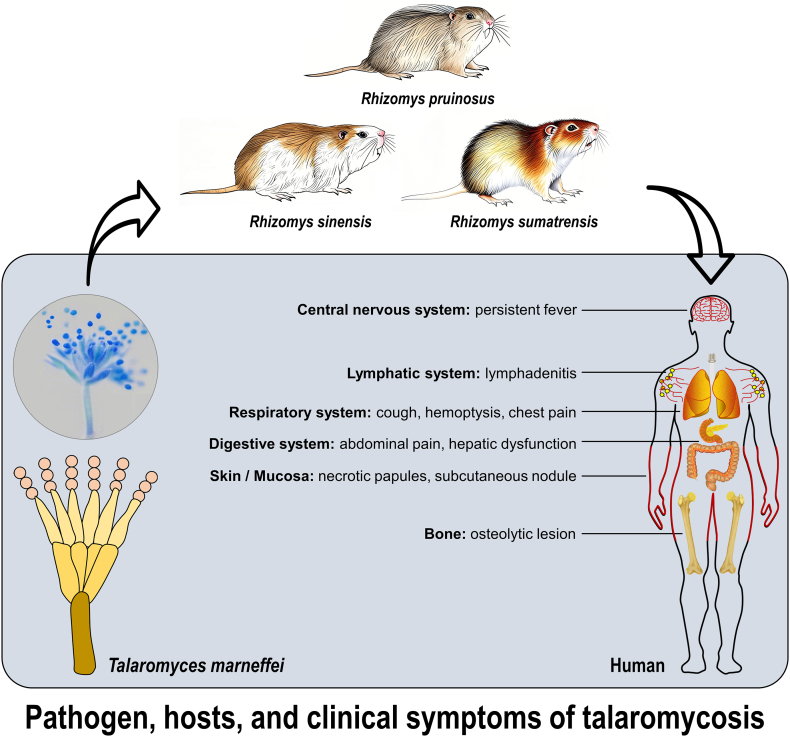
Pathogen, hosts and clinical symptoms of talaromycosis.

Based on the above challenges, in this study we first projected the risk distribution of *T. marneffei* and TSM across China under the One Health framework integrating fungal ecology, reservoir distribution, climate projections, and human vulnerability (Fig. 1). Collectively, the aims of this study are: (1) to model the current distribution of habitat suitability for the three key reservoir bamboo rat species (*R. pruinosus*, *R. sinensis*, *R. sumatrensis*) across China using a comprehensive set of environmental variables; (2) to identify the key environmental drivers determining the habitat distribution of three host species for *T. marneffei*; (3) to generate potential risk maps of human *T. marneffei* exposure by integrating the modeled reservoir suitability surfaces with socio-economic factors (e.g., HIV incidence, gross domestic product [GDP], human population density, etc.); and (4) to project potential distribution shifts under two representative climate change scenarios for the mid-(2060) and long-term (2100) future. Taken together, this integrated, multi-faceted approach represents a significant advancement in One Health-based prediction of *T. marneffei* and TSM risk in China. It advances beyond descriptive epidemiology by developing spatially explicit predictive tools capable of identifying both current high-risk zones and areas vulnerable to endemicity under projected climate and land-use scenarios. The resulting models and high-resolution risk maps are designed to directly inform public health decision-making, enabling targeted surveillance, enhanced diagnostic capacity, pre-emptive patient education, and optimized resource allocation in both endemic and emerging risk zones. Ultimately, the framework proposed in this study will contribute to reducing the substantial disease burden of this neglected mycosis.

## 2 Materials and methods

### 2.1 Species occurrence data

**Fig. 2:**
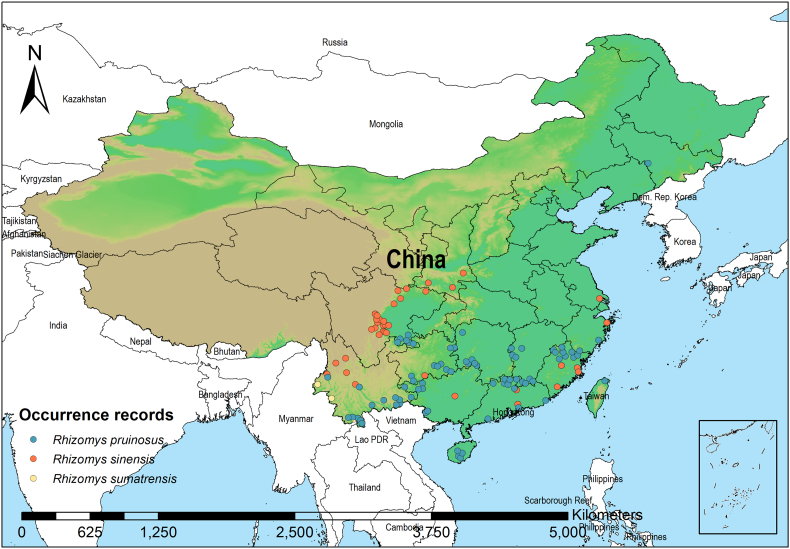
Species occurrence records of three animal hosts of *Talaromyces marneffei* in China.

A comprehensive and spatially accurate occurrence dataset of TM hosts is fundamental for robust ecological niche modeling. In this study, we compiled georeferenced records of *R. pruinosus*, *R. sinensis* and *R. sumatrensis* from three primary sources: (1) Database search from the Global Biodiversity Information Facility (GBIF; http://www.gbif.org) and the National Animal Collection Resource Center (NACRC; http://museum.ioz.ac.cn); (2) Literature review from CNKI (https://www.cnki.net) and PubMed (https://pubmed.ncbi.nlm.nih.gov) as of December 2023. The search strategy used a combination of keywords and subject terms related to the species names (“*Rhizomys pruinosus*”, “*Rhizomys sinensis*”, “*Rhizomys sumatrensis*”, “bamboo rat”), with a final inclusion of 4 articles (screening: *n* = 103; excluded: *n* = 99). The full search process is provided in Supplementary Fig. 1; and (3) Field investigation in epidemic regions in Sichuan and Yunnan Provinces. To mitigate spatial autocorrelation that can significantly bias model outputs and inflate performance estimates, we implemented spatial filtering using the “*Spatially Rarefy Occurrence Data*” tool within the species distribution model (SDM) toolbox in ArcGIS [36]. The detailed filtering parameters were as following - Input heterogeneity raster: Altitude_hetero_China.tif; Number of heterogeneity classes: 5; Classification type: natural_breaks; Maximum distance: 25 km; Minimum distance: 2 km. Finally, a total of 324 occurrence records for *R. pruinosus* (*n* = 178), *R. sinensis* (*n* = 118) and *R. sumatrensis* (*n* = 28) were compiled and used for further Maxent modeling (Fig. 2; Figs. S2-S4).

### 2.2 Environmental factors

The selection and processing of environmental variables significantly influence model performance and ecological interpretation. We sourced 104 high-resolution (30 arc-seconds, approximately 1 km at the equator) bioclimatic, topographic, and monthly environmental factors from WorldClim database (version 2.1; https://www.worldclim.org) (Table S1). Multicollinearity among environmental factors can destabilize model coefficients and obscure the interpretation of individual variable importance. To address this, we conducted a comprehensive pairwise Pearson correlation analysis (|*r*| ≥ 0.8 considered highly correlated). Within each highly correlated pair, we retained the variable with more biological relevance to *T. marneffei* ecology based on existing literature concerning thermal and moisture preferences of fungi and specifically *Talaromyces* species (Table S2). This process resulted in the selection of eleven key environmental factors for final model inclusion: Alt (elevation), Bio1 (annual mean temperature), Bio3 (isothermality), Bio7 (temperature annual range), Bio12 (annual precipitation), Bio14 (precipitation of the driest month), Bio15 (precipitation seasonality), Prec1 (average monthly precipitation in January), Prec7 (average monthly precipitation in July), Srad1 (average monthly solar radiation in January), and Wind6 (average monthly wind speed in June).

For future projections, corresponding layers for these identical factors were derived from the BCC-CSM2-MR global climate model outputs, which has good performance for future climate predictions in China, within the Coupled Model Intercomparison Project Phase 6 (CMIP6) ensemble [28,30,31]. These environmental layers were downscaled and bias-corrected to match WorldClim's resolution and format for the two representative SSP scenarios (SSP126, SSP585) across the four future time periods [37].

### 2.3 Socio-economic factors

Human infection risk of TM is intrinsically linked to the spatial coincidence of suitable fungal habitat and socio-economic conditions. In this study, socio-economic factors comprise three categories including HIV incidence, GDP, and human population density. HIV incidence data (2010−2020) were obtained from the National Health Commission (https://www.nhc.gov.cn). These data were first re-generated using the inverse distance weighting (IDW) tool in ArcGIS. The generated continuous surface representing the spatial gradient of exposure risk at the provincial level were then re-classified into different categories for subsequent risk calculation. GDP data were sourced from the National Bureau of Statistics for the year 2020 (https://www.stats.gov.cn), which were similarly converted and graded into spatial layers. Human population density data were acquired from WorldPop database (https://www.worldpop.org) for the year 2020, at a resolution of 30 arc-seconds (∼1 km at the equator). The raw density values (persons/km^2^) were then reclassified into ten ordinal classes following our previous protocols [31,32]. To ensure spatial consistency for integration with the environmental layers (30 arc-seconds), all socio-economic raster layers were resampled to a unified resolution of 30 arc-seconds using the IDW interpolation technique in ArcGIS. Collectively, the above socio-economic factors were then used for further integration with suitability distribution of TM hosts to generate TM/TSM risk distribution maps.

### 2.4 Modeling framework

**Fig. 3:**
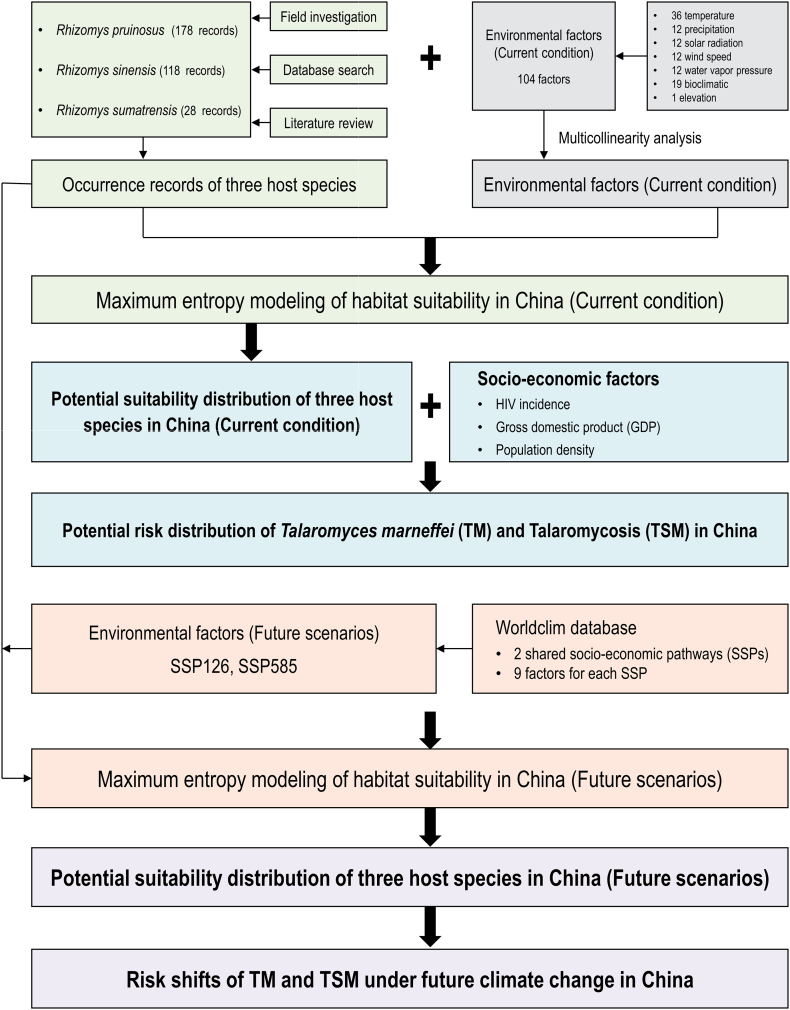
Experimental design of projecting risk distribution of *Talaromyces marneffei* and talaromycosis.

The modeling framework of this study consists of four parts as below (Fig. 3):

(1) Current suitability distribution of TM hosts: In this step, the potential geographic distribution of three TM animal hosts under current condition was predicted using Maxent model by integrating species occurrence records and environmental factors. The modeling output generated continuous probability maps representing the relative habitat suitability (ranging from 0 to 1) for each species. Based on this, the current suitability distribution pattern of TM hosts was shaped and used for further analysis.
(2) Environmental drivers of current distribution: Subsequently, the key environmental factors that affect the current distribution of TM hosts were investigated and analyzed. The percentage contribution results of Maxent modeling for each host species were used as reference to extract values of different suitability class. The environmental drivers of current distribution for TM hosts were then determined.
(3) TM/TSM risk distribution: This step integrated the suitability maps of TM hosts with socio-economic exposure factors (HIV incidence, GDP, human population density). The underlying hypothesis is that areas with high environmental suitability and high HIV incidence/human population density inherently present a greater risk of human-fungal contact and potential infection, acting as a proxy for factors like frequency of environmental exposure, land use patterns, and healthcare access limitations. HIV incidence, human population density, and GDP data were reclassified into ordinal categories representing relative exposure intensity. The infection risk index was calculated per grid cell by integrating the environmental suitability value and the reclassified values of socio-economic factors. This approach explicitly models the confluence of ecological niche and socio-economic factors as the primary determinants of TM/TSM transmission risks.
(4) Future distribution shifts: Finally, the future suitability shifts of TM hosts were projected under two shared socioeconomic pathway (SSP) scenarios – SSP126 (low radiative forcing) and SSP585 (very high) - representing divergent patterns of greenhouse gas emissions and socio-economic development. Projections were generated for four distinct 20-year periods spanning 2021–2100 (e.g., 2021–2040, 2041–2060, 2061–2080, 2081–2100). This step provided quantitative assessment of changes in the suitability/risk of “TM host - TM - TSM” as well as mechanistic insights into the drivers of projected change.

### 2.5 Maxent modeling and evaluation

The Maxent modeling was performed using the standalone software (version 3.3.3 k). Key settings for model calibration and execution were chosen based on best practices to optimize performance and avoid overfitting. A total of 100 bootstrap replicates were run. For each replicate, the occurrence data was randomly partitioned, with 75 % used for model training and the remaining 25 % reserved for independent testing. Model performance was evaluated using the area under the receiver operating characteristic curve (AUC/ROC). The mean test AUC across the 100 bootstrap replicates were used for model evaluation. To statistically assess whether model performance was significantly better than random, we generated 100 null models using the same environmental data but with randomly generated pseudo-absence points across the study area background. The AUC values of our actual models were then compared to the distribution of AUC values from these null models. A *P*-value <0.05 indicated significantly better performance than random expectation.

### 2.6 Suitability and risk classification

To facilitate interpretation and mapping of the continuous model outputs, both habitat suitability and infection risk were classified into different classes following our previous protocols [28,[30], [31], [32]]. Habitat suitability (T_s_): Low: T_s_ < 0.1; Moderate: 0.1 ≤ T_s_ < 0.3; High: 0.3 ≤ T_s_ < 0.5; Very high: T_s_ ≥ 0.5. Infection Risk (T_r_): Calculated per grid cell as: T_r_ = (T_s_) × (reclassified HIV incidence class value) × (reclassified GDP class value), for HIV-positive populations; Calculated per grid cell as: T_r_ = (T_s_) × (reclassified population density class value) × (reclassified GDP class value), for HIV-negative populations. To prevent overestimation of T_r_ values resulting from high HIV incidence or population density in specific regions, T_s_ values for low and medium classes are set to zero during the calculation. The resulting T_r_ values, reflecting the combined environmental and exposure risk, were then classified into the same four classes as T_s_: Low (T_r_ < 0.1), Moderate (0.1 ≤ T_r_ < 0.3), High (0.3 ≤ T_r_ < 0.5), and Very high (T_r_ ≥ 0.5). This standardized classification enables direct visual and quantitative comparison between purely environmental suitability and integrated human infection risk of TM.

### 2.7 Statistical analysis

Spatial data management, processing (spatial thinning, raster manipulation, reclassification, map algebra for risk calculation), and visualization were conducted using ArcGIS Desktop (version 10.8). Statistical analyses were performed using IBM SPSS Statistics (version 23.0) and GraphPad Prism (version 9.0).

## 3 Results

### 3.1 Robust habitat suitability predictions for TM host species using Maxent modeling

**Fig. 4:**
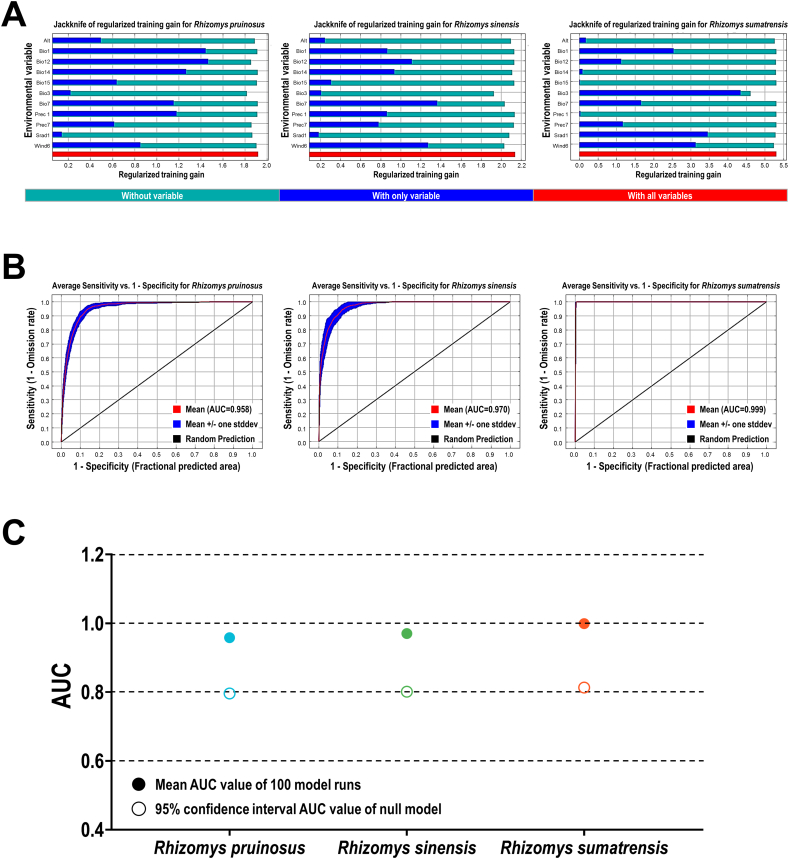
Jackknife training gain, ROC curves, AUC values and null-model validation for Maxent modeling of three TM hosts under current condition. (A): Jackknife results for *Rhizomys pruinosus*, *Rhizomys sinensis*, and *Rhizomys sumatrensis*, respectively. The Jackknife training gain for each TM host species was used as reference for generating percent contrition of environmental factors in Maxent modeling. (B): ROC curves and AUC values for *Rhizomys pruinosus*, *Rhizomys sinensis*, and *Rhizomys sumatrensis*, respectively. (C): Null-model validation of Maxent modeling for three TM hosts. TM: *Talaromyces marneffei*. ROC: receiver operating characteristic. AUC: area under the curve.

Maxent model analysis yielded robust and accurate predictions of habitat suitability distributions for the three TM host species, *Rhizomys pruinosus*, *Rhizomys sinensis*, and *Rhizomys sumatrensis*. Model performance was exceptionally high, as evidenced by Area Under the Curve (AUC) values for the test datasets ranging from 0.958 to 0.999. These values significantly exceeded the threshold for random prediction (95 % confidence interval), confirming the model's high reliability and its effectiveness in delineating the potential distribution patterns of these species (Fig. 4). Furthermore, the models demonstrated high predictive power with low omission rates. At the minimum training presence threshold, the mean omission rates on the test data were consistently low across the three species, indicating that the models successfully captured the fundamental environmental requirements of the hosts without being overly overfitted (Fig. S5). Jackknife analysis further elucidated the key environmental variables driving species distributions (Fig. 4), enhancing the ecological interpretability of the model outputs. Collectively, the Maxent models developed here provide a robust and reliable foundation for analyzing the current habitat suitability patterns of the three TM host species.

### 3.2 Heterogeneous habitat suitability concentrated in southern and southwestern China

**Fig. 5:**
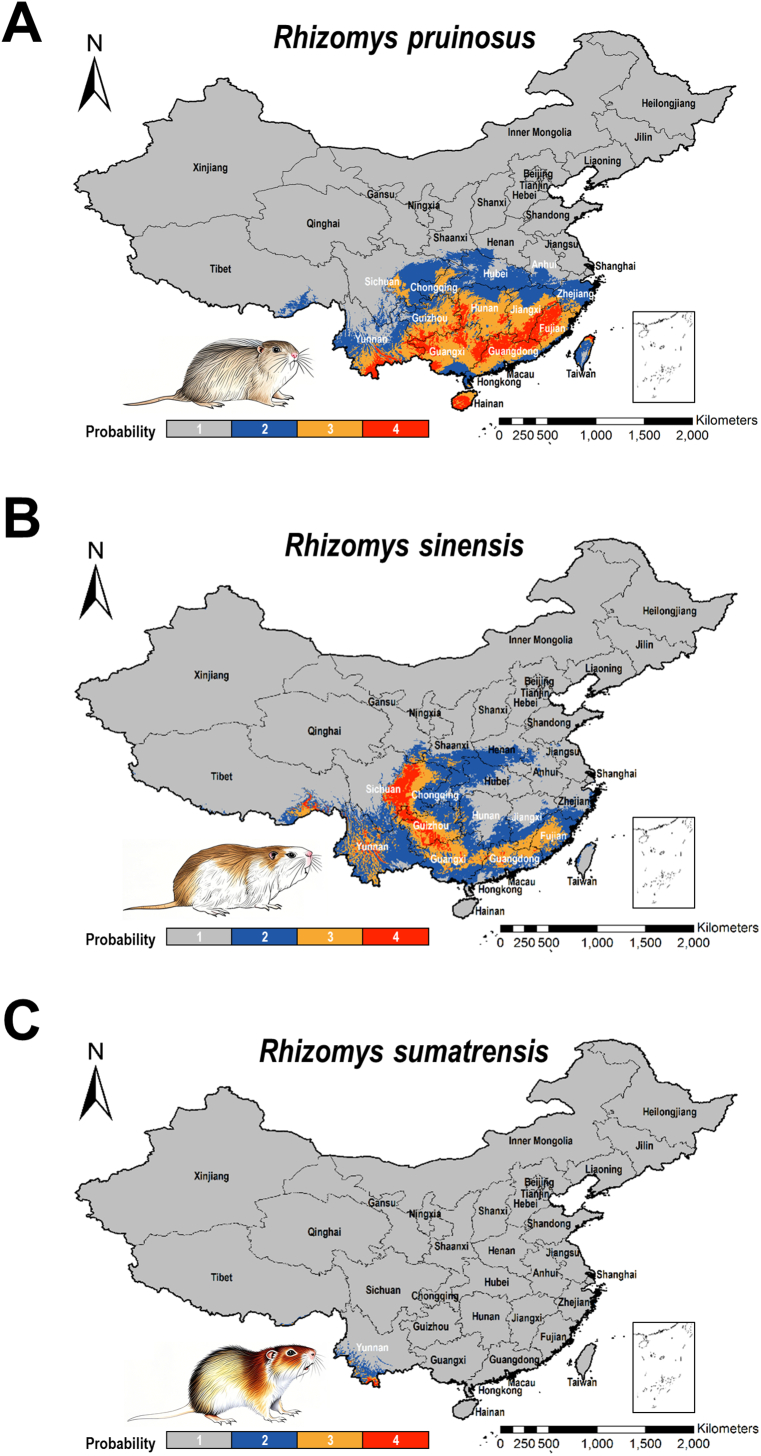
Potential suitability distribution of three TM hosts under current condition in China. The suitability distribution was represented as probability and divided into four classes. Class 1: Low suitability; Class 2: Moderate suitability; Class 3: High suitability; Class 4: Very high suitability. (A): *Rhizomys pruinosus*. (B): *Rhizomys sinensis*. (C): *Rhizomys sumatrensis*. TM: *Talaromyces marneffei*.

Spatial analysis revealed significant heterogeneity in habitat suitability distributions among the three TM host species. Specifically, high-suitability zones (suitability probability ≥0.5) for all species were predominantly concentrated within the historical TM endemic region between 20°N and 35°N latitude (Fig. 5; Table S3). *R. pruinosus* exhibited the broadest potential distribution. Its core high-suitability areas extensively covered southern and southeastern provinces, including Yunnan, Guizhou, Guangxi, Guangdong, Hunan, Jiangxi, Fujian, and Hainan (total suitable area: 972,586 km^2^; high suitability: 663,767 km^2^; very high suitability: 308,819 km^2^). *R. sinensis* displayed a more restricted and concentrated distribution. Core high-suitability areas were primarily located in Sichuan and Guizhou provinces, with additional scattered medium-to-high suitability patches in Yunnan, Guangxi, Guangdong, and Fujian (total suitable area: 607,535 km^2^; high suitability: 467,153 km^2^; very high suitability: 140,382 km^2^). *R. sumatrensis* exhibited the most limited distribution, with suitable areas predominantly scattered in southwestern Yunnan Province (total suitable area: 15,156 km^2^; high suitability: 11,319 km^2^; very high suitability: 3837 km^2^). These results demonstrate a pronounced spatial overlap and concentration of suitable habitats for all three TM host species in Southern and Southwestern China under current climate conditions, providing critical spatial data for targeting TM surveillance and control efforts.

### 3.3 Precipitation and temperature as dominant drivers of habitat suitability

Analysis of key environmental variables identified by the Maxent model and their response curves revealed that precipitation and temperature factors are the primary determinants of habitat suitability for TM host species, albeit with species-specific responses indicating niche differentiation (Table 1). For precipitation factors, annual precipitation (Bio12) was the dominant driver for both *R. pruinosus* (contribution: 46.4 %) and *R. sinensis* (contribution: 30.0 %). However, their optimal Bio12 ranges differed significantly (*R. pruinosus*: 1535.92 ± 226.78 mm; *R. sinensis*: 1145.03 ± 253.75 mm). Precipitation of the driest month (Bio14) was also a critical limiting factor for both species (*R. pruinosus*: contribution 31.3 %, optimum 32.38 ± 16.16 mm; *R. sinensis*: contribution 12.4 %, optimum 14.34 ± 6.90 mm), reinforcing the importance of precipitation seasonality. While for temperature factor, isothermality (Bio3) emerged as the dominant factor shaping the distribution of *R. sumatrensis* (contribution: 63.5 %, optimum: 52.8 ± 1.01), highlighting the significance of temperature stability for this species. Collectively, these findings highlight that the current habitat suitability patterns of TM host species are fundamentally constrained by climatic factors related to water availability and thermal regimes. The distinct optimal ranges and contribution rates of key variables for each species reflect their unique ecological niches and are coincident with known TM/TMS distribution patterns.

**Table 1:** Key environmental factors of each suitability class for suitability distribution of three host species.

***Rhizomys pruinosus***						
**Key environmental factor**	**Percent contribution**	**Low**	**Medium**	**High**	**Very high**	**Unit**
Bio12	46.4 %	380.02 ± 286.18	1321.71 ± 372.58	1470.35 ± 236.2	1535.92 ± 226.78	mm
Bio14	31.3 %	3.69 ± 5.21	23.64 ± 12.92	31.85 ± 12.68	32.38 ± 16.16	mm
Bio3	7.5 %	31.42 ± 6.12	30.87 ± 8.3	30.73 ± 6.72	33.83 ± 6.5	–
Bio1	4.0 %	3.95 ± 6.4	16.41 ± 2.61	17.81 ± 2.29	19.34 ± 1.95	°C
Alt	3.2 %	2063.37 ± 1772.57	719.57 ± 665.49	571.81 ± 448.51	447.45 ± 309.95	m
***Rhizomys sinensis***						
**Key environmental factor**	**Percent contribution**	**Low**	**Medium**	**High**	**Very high**	**Unit**
Bio12	30.0 %	427.26 ± 391.18	1248.73 ± 355.18	1329.48 ± 336.12	1145.03 ± 253.75	mm
Wind6	26.9 %	3.19 ± 0.79	2.02 ± 0.46	1.74 ± 0.32	1.53 ± 0.27	m/s
Bio14	12.4 %	5.43 ± 10	21.91 ± 13.75	23.27 ± 12.17	14.34 ± 6.9	mm
Bio3	10.7 %	30.98 ± 5.96	33.26 ± 8.16	34.22 ± 7.7	31.78 ± 5.93	–
Bio7	10.4 %	42.29 ± 8.33	26.81 ± 4.06	25.58 ± 2.17	26.14 ± 1.3	°C
***Rhizomys sumatrensis***						
**Key environmental factor**	**Percent contribution**	**Low**	**Medium**	**High**	**Very high**	**Unit**
Bio3	63.5 %	31.29 ± 6.26	49.94 ± 2.44	50.65 ± 2.61	52.8 ± 1.01	–
Wind6	10.2 %	2.97 ± 0.88	1.33 ± 0.16	1.26 ± 0.14	1.31 ± 0.08	m/s
Alt	9.7 %	1796.17 ± 1715.08	1192.68 ± 323.6	936.33 ± 307.02	765.86 ± 133.67	m
Srad1	8.2 %	8851.55 ± 1817.74	14,629.65 ± 872.39	14,851.13 ± 686.08	14,331.21 ± 430.22	kJ/m^2^/day
Bio1	6.2 %	6.4 ± 7.84	19.7 ± 1.17	21.05 ± 1.12	22.1 ± 0.59	°C

*Note*: The first five key environmental factors for each host species of TM were shown in table. The full name for each factor was: Alt - Altitude; Bio1 - Annual mean temperature; Bio3 - Isothermality; Bio7 - Temperature annual range; Bio12 - Annual precipitation; Bio14 - Precipitation of driest month; Srad1 - Solar radiation of January; Wind6 - Wind speed of June.

### 3.4 TM/TSM transmission risk map revealed an overlapping yet more precise distribution relative to host suitability distribution

**Fig. 6:**
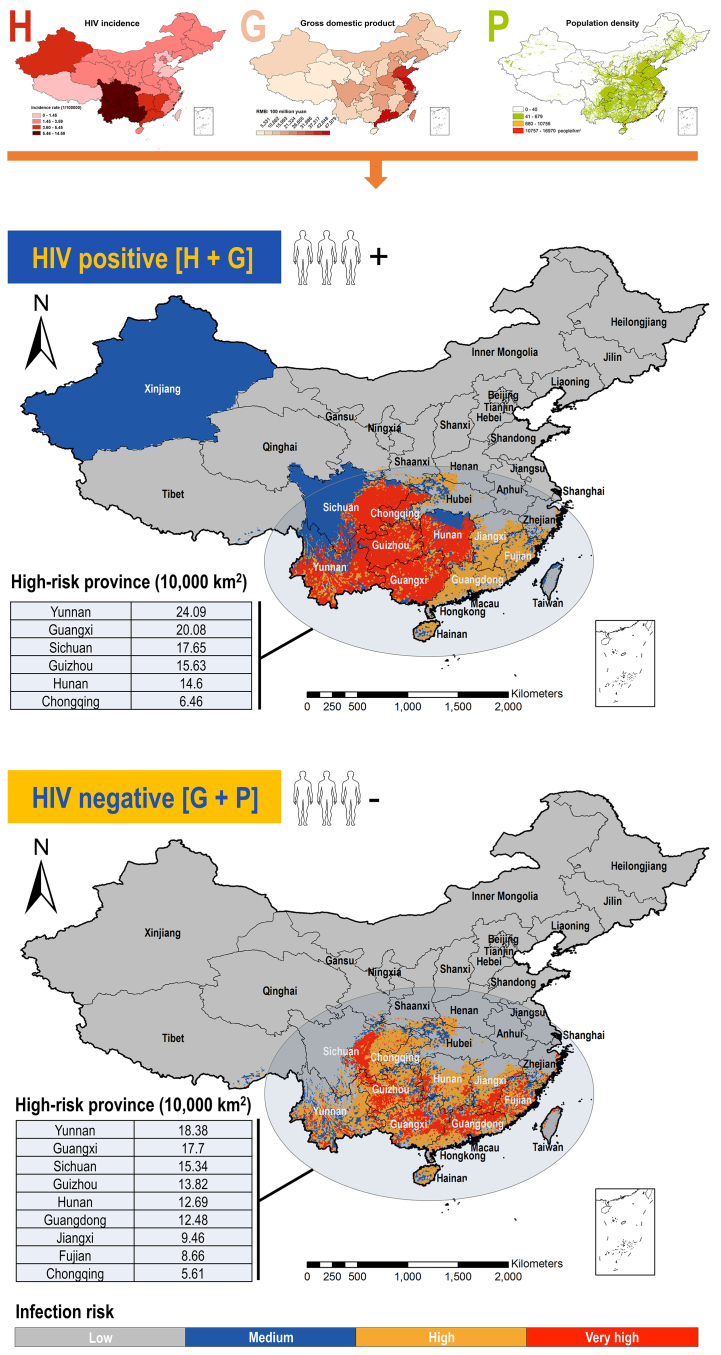
Potential risk distribution of TM/TSM under current condition in China. The risk distribution was calculated based on suitability maps and socio-economic (HIV incidence + GDP, for HIV-positive populations; Population density + GDP, for HIV-negative populations) maps and divided into four classes. Class 1: Low risk; Class 2: Moderate risk; Class 3: High risk; Class 4: Very high risk. The area of high-risk ([High + Very high] classes) provinces were calculated and given in tables. TM: *Talaromyces marneffei*. TSM: talaromycosis.

Integrating host habitat suitability with socio-economic factors (HIV incidence, human population density, GDP) generated spatially refined maps of TM/TSM transmission risk hotspots in China, stratified by HIV status (Fig. 6; Table 2). Overall, the spatial distribution of transmission risk exhibited a high degree of concordance with the host habitat suitability zones, being heavily concentrated in Southern and Southwestern China. However, the inclusion of socio-economic factors conferred significantly higher spatial resolution and accuracy to the risk model compared to the host suitability model alone. For HIV-positive high-risk areas, these were intensely concentrated in Yunnan (240,851 km^2^ of [High + Very high] classes; the same below), Guangxi (200,833 km^2^), Sichuan (176,458 km^2^), Guizhou (156,250 km^2^), Hunan (146,007 km^2^), and Chongqing (64,566 km^2^). This distribution is coincident closely with regions reporting high clinical incidence rates of TM/TSM. While for HIV-negative high-risk areas, distribution was broader but still focused, covering Yunnan (183,767 km^2^), Guangxi (176,962 km^2^), Sichuan (153,403 km^2^), Guizhou (138,229 km^2^), Hunan (126,858 km^2^), Guangdong (124,826 km^2^), Jiangxi (94,566 km^2^), Fujian (86,597 km^2^), and Chongqing (56,128 km^2^). Given the rising incidence of HIV-negative TSM cases in recent years, populations in these regions warrant heightened vigilance. Taken together, the above results confirm a strong spatial association between TM/TSM transmission risk and host habitat suitability. Specifically, the integration of socio-economic data enabled a more precise delineation of risk hotspots, providing essential granularity for targeted public health interventions.

**Table 2:** High-risk regions of *Talaromyces marneffei* and talaromycosis for HIV-positive and HIV-negative population in China.

**High-risk region**	**Low**	**Medium**	**High**	**Very high**	**[High + Very high]**
*HIV-positive population*					
Yunnan	0.0712	9.6927	5.9583	18.1267	24.0851
Guangxi	0.0035	0.6424	2.7205	17.3628	20.0833
Sichuan	0.4149	27.4479	2.3108	15.3351	17.6458
Guizhou	0	0.3559	1.7795	13.8455	15.625
Hunan	0.1736	4.6285	2.0469	12.5538	14.6007
Chongqing	0.0712	1.2014	0.8802	5.5764	6.4566
China	588.816	228.0243	55.4097	82.9184	138.3281
*HIV-negative population*					
Yunnan	9.8420	6.0417	14.5226	3.8542	18.3767
Guangxi	0.6615	2.5660	10.0313	7.6649	17.6962
Sichuan	27.8333	2.3351	9.7326	5.6076	15.3403
Guizhou	0.3576	1.8003	8.1389	5.6840	13.8229
Hunan	4.7674	1.9497	10.2622	2.4236	12.6858
Guangdong	1.9826	0.9931	5.8594	6.6233	12.4826
Jiangxi	4.8073	1.0122	6.6719	2.7847	9.4566
Fujian	0.4010	1.7361	5.3785	3.2813	8.6597
Chongqing	1.2309	0.8854	5.2066	0.4063	5.6128
China	812.5434	24.0938	85.6059	39.5069	125.1128

*Note*: Area of main high-risk provinces for HIV-positive and HIV-negative population was calculated and shown in table. Unit: 10,000 km^2^.

### 3.5 Divergent responses of host habitat suitability to future climate change

**Fig. 7:**
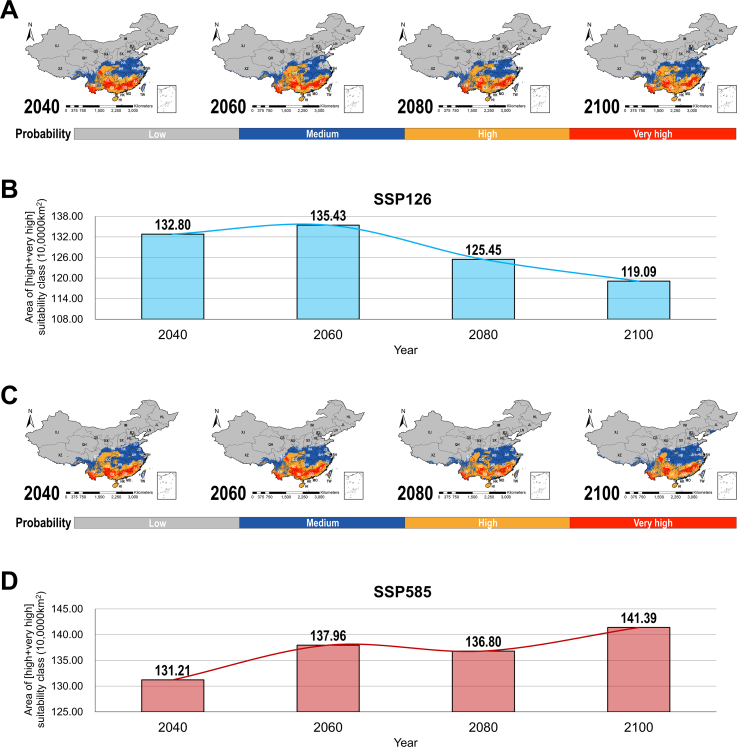
Suitability shifts of three TM hosts under future scenarios. (A) - (B): Integrated suitability shifts of three TM hosts under SSP126, from 2021 to 2100. (C) - (D): Integrated suitability shifts of three TM hosts under SSP585, from 2021 to 2100. The areas were calculated as high and very high suitable regions. TM: *Talaromyces marneffei*. SSP: shared socio-economic pathway. The abbreviations for each province are as follows: AH - Anhui; BJ - Beijing; CQ - Chongqing; FJ - Fujian; GS - Gansu; GD - Guangdong; GX - Guangxi; GZ - Guizhou; HI - Hainan; HE - Hebei; HA - Henan; HL - Heilongjiang; HB - Hubei; HN - Hunan; JL - Jilin; JS - Jiangsu; JX - Jiangxi; LN - Liaoning; IM - Inner Mongolia; NX - Ningxia; QH - Qinghai; SD - Shandong; SX - Shanxi; SN - Shaanxi; SH - Shanghai; SC - Sichuan; TJ - Tianjin; XZ - Tibet; XJ - Xinjiang; YN - Yunnan; ZJ - Zhejiang; HK - Hong Kong; MO - Macao; TW - Taiwan.

Projections under different shared socioeconomic pathway (SSP) scenarios revealed divergent patterns for host habitat suitability in response to future climate change, with variations depending on both host species and emission intensity (Fig. 7). For low emission scenario (SSP126), the total composite suitable area for the three host species exhibited an overall declining trend. After a slight increase from 1,327,969 km^2^ (area of [High + Very high] classes, the same below; 2040s) to 1,354,323 km^2^ (2060s), suitability areas contracted to 1,254,462 km^2^ (2080s) and further to 1,190,903 km^2^ (2100 s). While for high emission scenario (SSP585), in contrast, the total composite suitable area showed an increasing trend under high emissions. Area expanded from 1,312,083 km^2^ (2040s) to 1,379,601 km^2^ (2060s), remained relatively stable at 1,367,986 km^2^ (2080s), and further increased to 1,413,924 km^2^ (2100 s). Collectively, these results demonstrate that habitat suitability for TM host species exhibits differentiated and pathway-dependent responses to climate change. The direction and magnitude of change are critically influenced by future greenhouse gas emission patterns. This highlights the necessity of incorporating dynamic host distribution shifts under diverse climate change scenarios into future TM/TSM risk assessments and prevention strategies to ensure their long-term adaptability and effectiveness.

## 4 Discussion

In general, we first provide high-resolution, statistically robust predictions of habitat suitability for the three epidemiologically critical TM host species - *R. pruinosus*, *R. sinensis*, and *R. sumatrensis*. Specifically, our modeling outputs reveal a significant concentration of high-suitability regions for all three species within Southern and Southwestern China (20°N-35°N), coincident with the historical TM/TSM endemic zones. This finding corroborates large-scale epidemiological zonation proposed by our group but delivers transformative granularity [28]. Previous efforts in TM/TSM transmission take “bamboo rats” as a single functional unit, obscuring critical species-level heterogeneity [26]. Our species-specific approach unveils this complexity: *R. pruinosus* dominates southeastern provinces with extensive high-suitability areas (308,819 km^2^), *R. sinensis* exhibits a Sichuan-Guizhou core (high-suitability area of 140,382 km^2^) with fragmented eastern outliers, and *R. sumatrensis* is tightly constrained to southwestern Yunnan (high-suitability area of 3837 km^2^). Quantifying these areas provides the first comprehensive spatial baseline for main TM hosts in China. The significant interspecies overlap (> 70 % co-occurrence in core grids) within this latitudinal belt starkly contrasts with the more dispersed patterns predicted for rodent hosts in northern China in previous study, highlighting the unique biogeographical constraints shaping TM enzooticity [38]. This refined spatial partitioning identifies not just “Southern China” as risky, but pinpoints multi-species hotspot provinces (e.g., Yunnan, Guizhou, and Guangxi) where surveillance should be most intensive due to amplified host biomass and potential pathogen transmission interfaces.

Our analysis on environmental drivers identifies precipitation (Bio12, Bio14) and temperature (Bio3) as the dominant factors shaping host distributions. This reinforces the foundational work of previous studies on bamboo rat physiology, which identified moisture sensitivity and thermoregulatory constraints as key survival factors [18,39]. However, we reveal profound niche differentiation previously obscured. While previous studies suggested general moisture dependencies for *Rhizomys* species, our response curves and variable contributions demonstrate distinct hydrological optima. *R. pruinosus* thrives in regions with higher annual precipitation (optimal Bio12: ∼1500 mm) and tolerates drier dry seasons (Bio14 optimum: ∼32 mm), explaining its prevalence in humid southeastern coasts and Hainan. Conversely, *R. sinensis*, while also precipitation-driven, prefers slightly lower annual totals (∼1100 mm) and crucially requires lower dry-season moisture (Bio14 optimum: ∼14 mm), coincident with its core distribution in Sichuan/Guizhou regions. *R. sumatrensis* diverges fundamentally, governed primarily by isothermality (Bio3 > 60 %; optimum ∼52), reflecting its adaptation to the thermally stable, low-seasonality environments of southern Yunnan's tropical montane forests. This temperature stability requirement, potentially linked to reproductive phenology or fungal symbiont survival, was not emphasized in previous studies [18]. Collectively, this niche partitioning has direct epidemiological significance. The distinct moisture niches of *R. pruinosus* and *R. sinensis* possibly correlate with regional TM strain variations, suggesting co-evolutionary pathways. Furthermore, *R. sumatrensis*'s confinement to stable thermal regimes makes it particularly vulnerable to climate variability, potentially explaining its role in sporadic, localized outbreaks. These findings provide mechanistic hypotheses linking climate gradients to host fitness and, by extension, pathogen maintenance capacity. This correlation further challenges simplistic “moisture - risk” models and necessitates species-tailored ecological understanding for predictive epidemiology.

The integration of high-fidelity host suitability maps with spatially explicit socio-economic drivers (HIV incidence, population density, GDP) represents a paradigm shift in TM/TSM risk mapping. While previous foundational studies established the link between host presence and human cases, and mapped human vulnerability factors, our model is the first to dynamically couple these dimensions across China at this resolution [[40], [41], [42]]. The resulting risk maps exhibit significant concordance with the host core zones, reaffirming the host's ecological primacy. Critically, however, incorporating socio-economics drastically refined risk delineation beyond what host suitability alone could achieve [43]. This integrated approach had significant improvement for TM/TSM risk prediction. For HIV-positive populations, we identified hyper-endemic foci (e.g., Yunnan-Guangxi border) coincident perfectly with historical TSM epicenters, validating the model retrospectively [17]. More importantly, for the increasing caseload of TM/TSM in HIV-negative populations, we reveal extensive, previously underappreciated risk zones (e.g., 124,826 km^2^ in Guangdong, 94,566 km^2^ in Jiangxi) - areas where traditional surveillance focused on historical HIV-positive hotspots might miss emerging threats [16,44]. Our quantification of these areas (total high-risk region: ∼1.2 million km^2^) provides the first empirical basis for reallocating surveillance resources. The model also elucidates why risk extends beyond core host zones. High population density (> 300 persons/km^2^) and poor healthcare access (> 1 h travel time to tertiary care) in provinces like Fujian and Jiangxi amplify spillover potential even where host suitability is moderate. Taken together, our stratification by HIV status is operationally critical. HIV-positive hotspots demand enhanced diagnostics and antifungal stockpiling (e.g., liposomal amphotericin B), while HIV-negative zones require community awareness campaigns on early symptom recognition and environmental exposure reduction (e.g., bamboo rat handling practices). This dual-strategy approach, enabled by our integrated maps, offers a blueprint for precision public health in the future.

Our projections under SSP scenarios reveal that the future of TM host suitability – and thus disease risk - is not monolithic but critically dependent on both emission pathways and host species traits. Projections under the low-emission scenario (SSP126) revealed an overall contraction trend, with the total composite suitable area for the three host species decreasing by approximately 10 % (from ∼1.33 million km^2^ to ∼1.19 million km^2^) by the end of the century. This trend is consistent with general predictions of climate-induced range loss for small mammals [45]. In stark contrast, under the high-emission scenario (SSP585), the suitable area expanded by approximately 8 % (from ∼1.31 million km^2^ to ∼1.41 million km^2^) by 2100, presenting a counterintuitive and novel risk paradigm. This expansion is driven primarily by the colonization of *R. pruinosus* and *R. sinensis* in warmer, wetter habitats in Central China (e.g., Hubei, Henan), as reported in recent years [46]. The physiological plasticity of the two *Rhizomys* species, evidenced by their broad current niche, likely underpins this adaptability - a trait less pronounced in the more stenotopic *R. sumatrensis*. Collectively, our results challenge the long-term validity of current control zones defined by historical incidence. Specifically, the divergent patterns under SSP126 vs. SSP585 quantify the disease burden implications of climate policy. It is estimated that high emissions could expand the population living in high-risk zones by an estimated 50–70 million by 2100. This demands proactive adaptation: surveillance networks must become dynamic, incorporating real-time climate data and predictive modeling to track host range shifts. Vaccine development should also prioritize strains associated with expansionist hosts like *R. pruinosus*. Ultimately, our findings position TM/TSM not just as a current health challenge, but as a climate-sensitive barometer - where mitigation action directly curtails future zoonotic risk amplification.

In addition, our study has several limitations that should be considered when interpreting the results. Firstly, our future projections rely on a single global climate model (GCM), BCC-CSM2-MR, and two contrasting climate scenarios (SSP126 and SSP585). While the selected GCM performs well in simulating East Asian climate patterns, and the use of extreme scenarios effectively brackets the potential range of future climate impacts, this approach does not capture the full uncertainty inherent in climate projections [28,32]. The inclusion of a multi-model ensemble and intermediate scenarios (e.g., SSP245) would provide a more robust and probabilistic assessment of future risk, representing a critical avenue for future research. Secondly, while the high AUC values indicate excellent model performance, our model evaluation primarily relied on this threshold-independent metric. A more comprehensive assessment, incorporating metrics such as partial ROC, and AICc to better evaluate model fit, predictive power, and parsimony, would further strengthen the ecological niche modeling framework. Addressing these aspects in subsequent studies will be essential for refining predictive accuracy and uncertainty quantification in the face of climate change.

## Author contributions

NX and BC conceived the study. XM, KW, and TL collected data. NX, KW and BC analyzed the data. NX wrote the first draft of the manuscript. All authors contributed to the interpretation of the results and edited the manuscript.

## Funding

This work was supported by the Natural Science Basic Research Program of Shaanxi Province, China [2023-JC-QN-0858], the Free Exploration Program of the Second Affiliated Hospital, 10.13039/100008235School of Medicine, 10.13039/501100002412Xi'an Jiaotong University [2020YJ(ZYTS)605], and the 10.13039/501100001809National Natural Science Foundation of China [81900620].

## Ethics approval and consent to participate

Not applicable.

## Consent for publication

All authors acknowledge the submission and approve the manuscript for publication.

## Clinical trial number

Not applicable.

## CRediT authorship contribution statement

**Nan Xu:** Writing – original draft, Resources, Investigation, Formal analysis. **Xiaoyun Min:** Resources. **Kunyi Wu:** Resources, Investigation. **Ting La:** Software, Resources. **Bo Cao:** Writing – review & editing, Visualization, Supervision, Formal analysis, Conceptualization.

## Declaration of competing interest

The authors declare no conflicts of interest.

## Data Availability

Data will be made available on request.

## Appendix A Supplementary data

Supplementary material 1 Image 1

Supplementary material 2 Image 2
