# Supplementary material for: Environmental transmission from bamboo rats: Mapping current and future talaromycosis risk in China under climate change
Source: One Health. 2025 Nov 1;21:101264. doi: 10.1016/j.onehlt.2025.101264 (PMC12639262; doi:10.1016/j.onehlt.2025.101264)
Supplement: Supplementary file 1 — Supplementary material 1 [file mmc1.docx]

**Supplementary Information for**

Environmental transmission from bamboo rats: Mapping current and future talaromycosis risk in China under climate change

Nan Xu^1,2^, Xiaoyun Min^3^, Kunyi Wu^3^, Ting La^4^, Bo Cao^2,3,5,6*^

^1^ Department of Clinical Laboratory, The Second Affiliated Hospital of Xi’an Jiaotong University, Xi’an 710004, China

^2^ Department of Infectious Diseases, The Second Affiliated Hospital of Xi’an Jiaotong University, Xi’an 710004, China

^3^ Core Research Laboratory, The Second Affiliated Hospital of Xi’an Jiaotong University, Xi’an 710004, China

^4^ National-Local Joint Engineering Research Center of Biodiagnosis & Biotherapy, The Second Affiliated Hospital of Xi’an Jiaotong University, Xi’an 710004, China

^5^ College of Life Sciences, Shaanxi Normal University, Xi’an 710119, China

^6^ Key Laboratory of Surgical Critical Care and Life Support (Xi'an Jiaotong University), Ministry of Education, Xi'an, 710004, China

***Correspondence to**:

Bo Cao, Ph.D., Professor

The Second Affiliated Hospital of Xi’an Jiaotong University

Xi’an 710004, China

Email: [bo_cao@xjtu.edu.cn](mailto:bo_cao@xjtu.edu.cn); [bocao@snnu.edu.cn](mailto:bocao@snnu.edu.cn)

**This file includes:**

*Supplementary Figure 1-5*

*Supplementary Table 1,3,4*


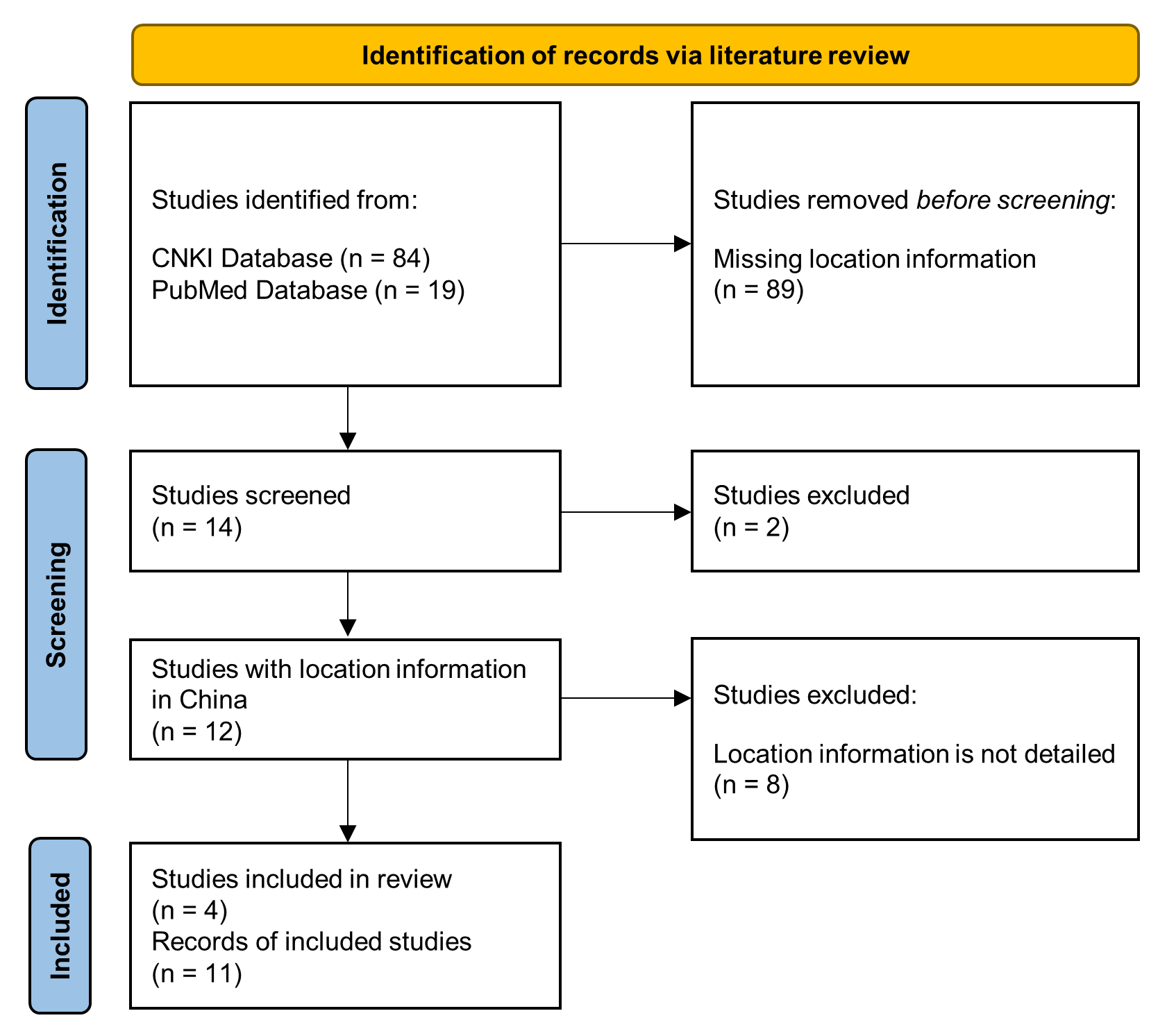


**Figure S1. PRISMA flow diagram for the identification of occurrence records via literature review in this study.**


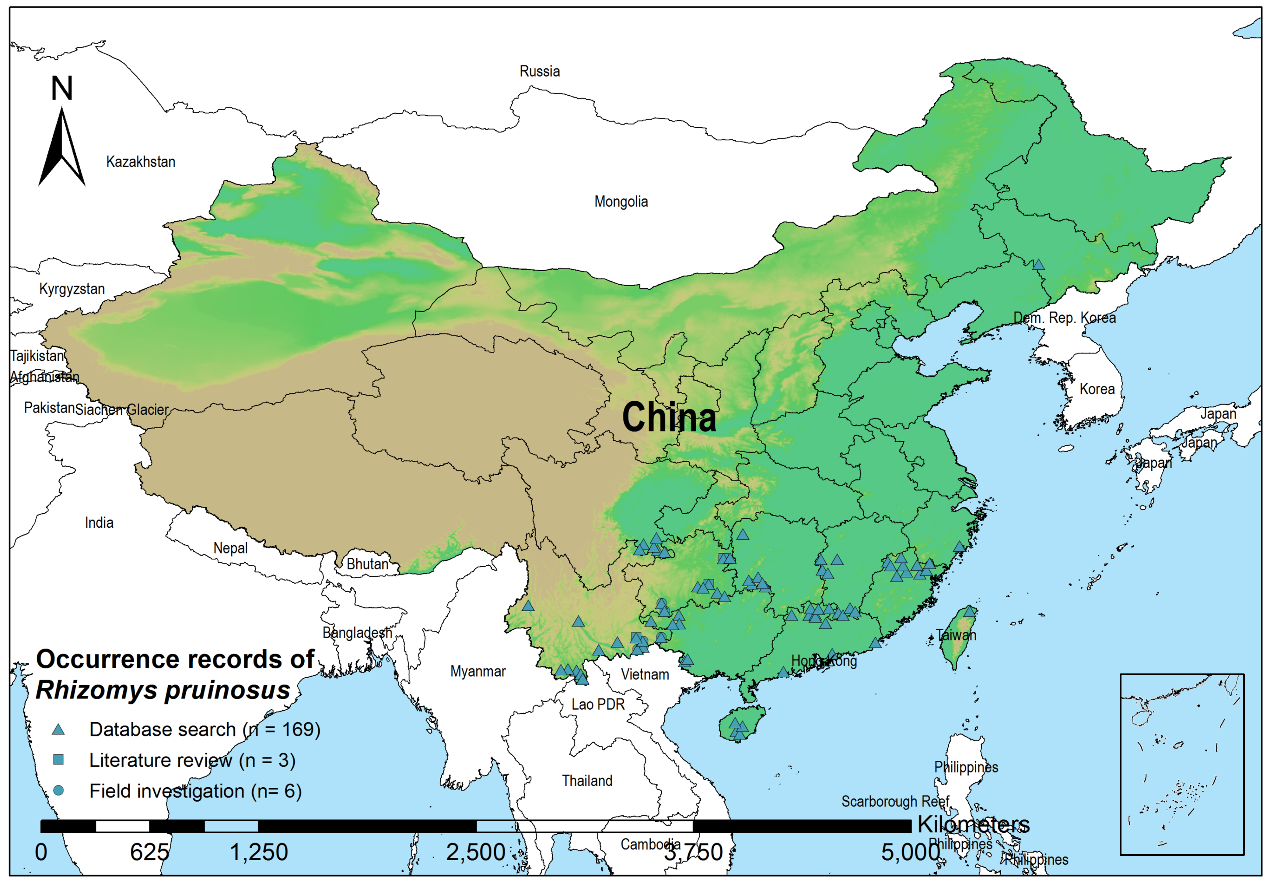


**Figure S2: Species occurrence records of *Rhizomys pruinosus* in China.**


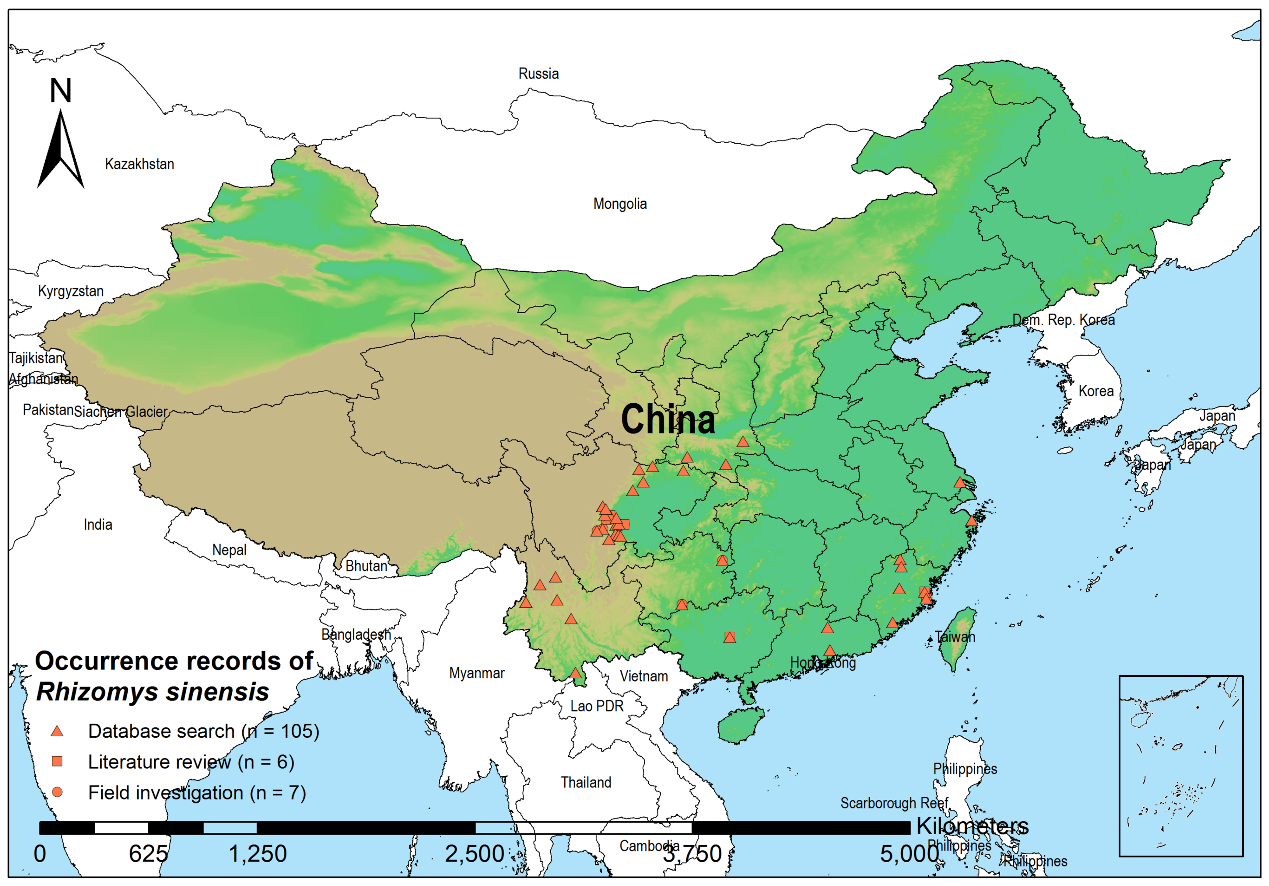


**Figure S3: Species occurrence records of *Rhizomys sinensis* in China.**


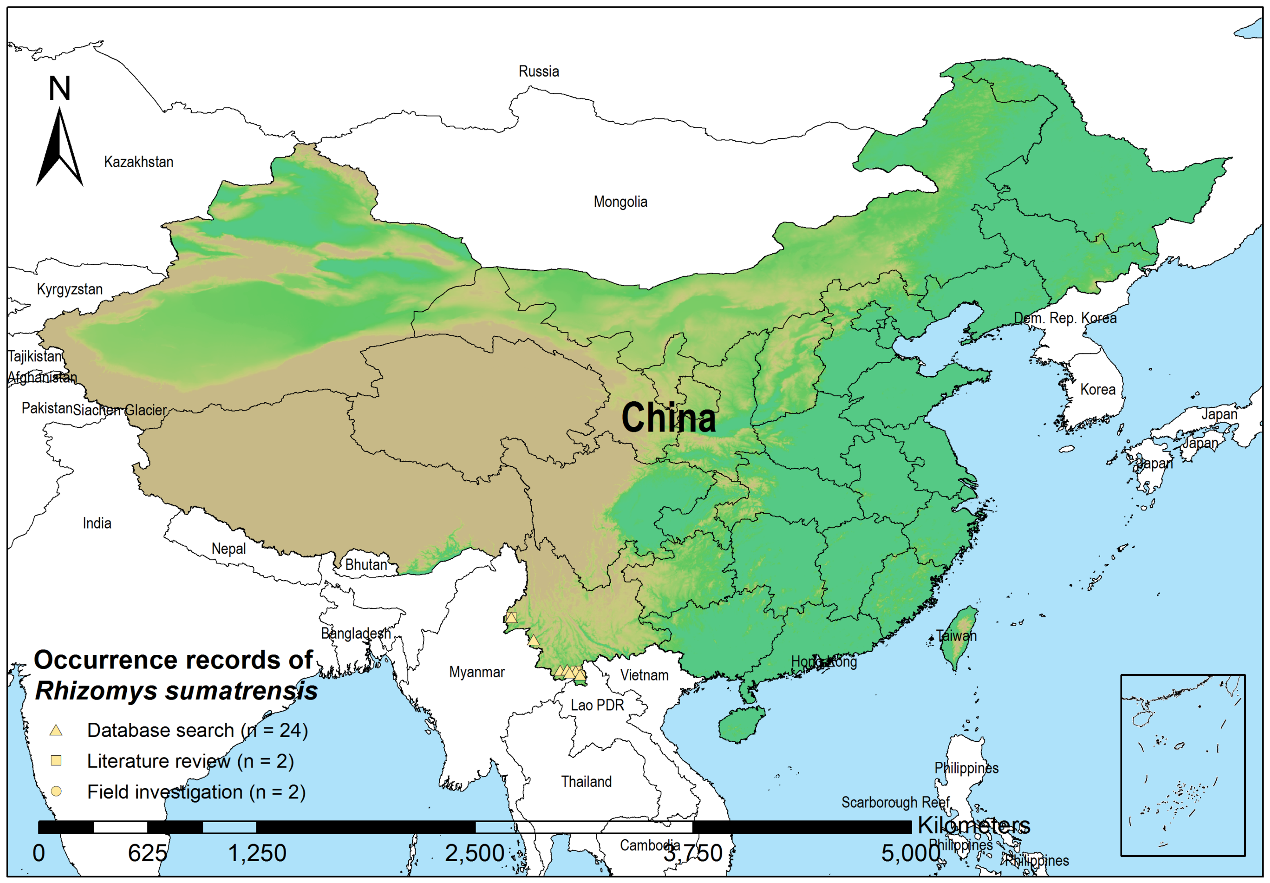


**Figure S4: Species occurrence records of *Rhizomys sumatrensis* in China.**


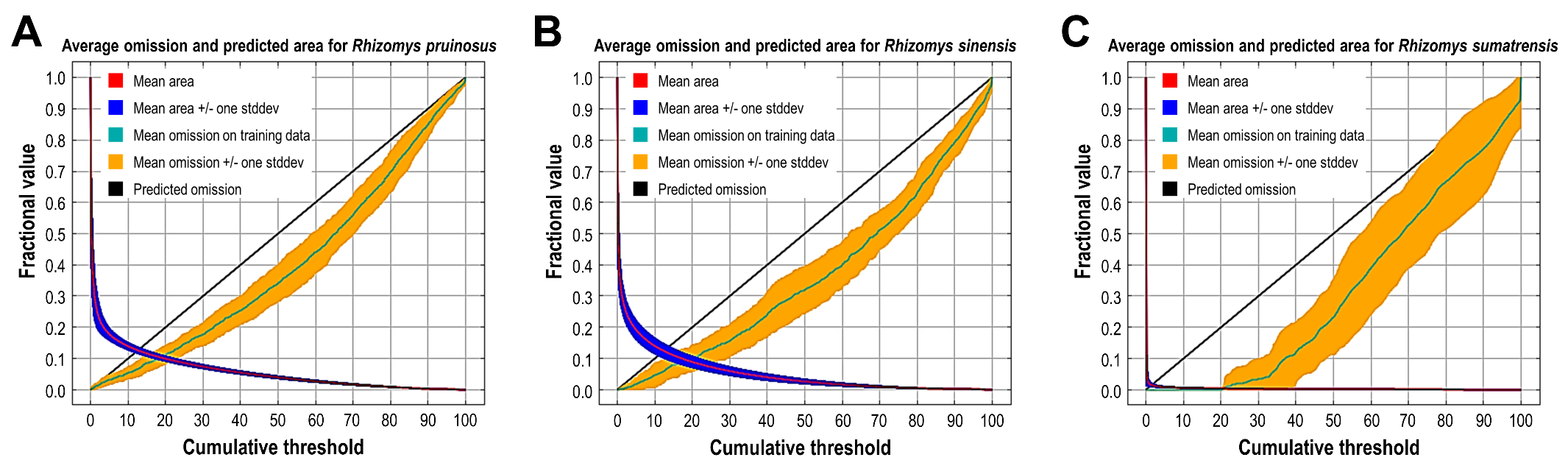


**Figure S5: Omission validation for Maxent modeling of three hosts of *Talaromyces marneffei* under current condition.**

**Table S1.** Environmental factors used in preliminary analysis in this study.

| **Variable** | **Description** | **Unit** |
| --- | --- | --- |
| Alt | altitude | m |
| Bio1 | annual mean temperature | °C |
| Bio2 | mean diurnal range (mean of monthly (max temp - min temp)) | °C |
| Bio3 | isothermality (bio2/bio7) (×100) | - |
| Bio4 | temperature seasonality (standard deviation ×100) | - |
| Bio5 | max temperature of warmest month | °C |
| Bio6 | min temperature of coldest month | °C |
| Bio7 | temperature annual range (bio5-bio6) | °C |
| Bio8 | mean temperature of wettest quarter | °C |
| Bio9 | mean temperature of driest quarter | °C |
| Bio10 | mean temperature of warmest quarter | °C |
| Bio11 | mean temperature of coldest quarter | °C |
| Bio12 | annual precipitation | mm |
| Bio13 | precipitation of wettest month | mm |
| Bio14 | precipitation of driest month | mm |
| Bio15 | precipitation seasonality (coefficient of variation) | - |
| Bio16 | precipitation of wettest quarter | mm |
| Bio17 | precipitation of driest quarter | mm |
| Bio18 | precipitation of warmest quarter | mm |
| Bio19 | precipitation of coldest quarter | mm |
| Prec1 | precipitation of January | mm |
| Prec2 | precipitation of February | mm |
| Prec3 | precipitation of ‌March‌ | mm |
| Prec4 | precipitation of ‌April‌ | mm |
| Prec5 | precipitation of May | mm |
| Prec6 | precipitation of ‌June | mm |
| Prec7 | precipitation of ‌July‌ | mm |
| Prec8 | precipitation of ‌August‌ | mm |
| Prec9 | precipitation of ‌September‌ | mm |
| Prec10 | precipitation of ‌October‌ | mm |
| Prec11 | precipitation of ‌November‌ | mm |
| Prec12 | precipitation of ‌December‌ | mm |
| Srad1 | solar radiation of January | kJ/m^2^/day |
| Srad2 | solar radiation of February | kJ/m^2^/day |
| Srad3 | solar radiation of ‌March‌ | kJ/m^2^/day |
| Srad4 | solar radiation of ‌April‌ | kJ/m^2^/day |
| Srad5 | solar radiation of May | kJ/m^2^/day |
| Srad6 | solar radiation of ‌June | kJ/m^2^/day |
| Srad7 | solar radiation of ‌July‌ | kJ/m^2^/day |
| Srad8 | solar radiation of ‌August‌ | kJ/m^2^/day |
| Srad9 | solar radiation of ‌September‌ | kJ/m^2^/day |
| Srad10 | solar radiation of ‌October‌ | kJ/m^2^/day |
| Srad11 | solar radiation of ‌November‌ | kJ/m^2^/day |
| Srad12 | solar radiation of ‌December‌ | kJ/m^2^/day |
| Tavg1 | average temperature of January | °C |
| Tavg2 | average temperature of February | °C |
| Tavg3 | average temperature of ‌March‌ | °C |
| Tavg4 | average temperature of ‌April‌ | °C |
| Tavg5 | average temperature of May | °C |
| Tavg6 | average temperature of ‌June | °C |
| Tavg7 | average temperature of ‌July‌ | °C |
| Tavg8 | average temperature of ‌August‌ | °C |
| Tavg9 | average temperature of ‌September‌ | °C |
| Tavg10 | average temperature of ‌October‌ | °C |
| Tavg11 | average temperature of ‌November‌ | °C |
| Tavg12 | average temperature of ‌December‌ | °C |
| Tmax1 | maximum temperature of January | °C |
| Tmax2 | maximum temperature of February | °C |
| Tmax3 | maximum temperature of ‌March‌ | °C |
| Tmax4 | maximum temperature of ‌April‌ | °C |
| Tmax5 | maximum temperature of May | °C |
| Tmax6 | maximum temperature of ‌June | °C |
| Tmax7 | maximum temperature of ‌July‌ | °C |
| Tmax8 | maximum temperature of ‌August‌ | °C |
| Tmax9 | maximum temperature of ‌September‌ | °C |
| Tmax10 | maximum temperature of ‌October‌ | °C |
| Tmax11 | maximum temperature of ‌November‌ | °C |
| Tmax12 | maximum temperature of ‌December‌ | °C |
| Tmin1 | minimum temperature of January | °C |
| Tmin2 | minimum temperature of February | °C |
| Tmin3 | minimum temperature of ‌March‌ | °C |
| Tmin4 | minimum temperature of ‌April‌ | °C |
| Tmin5 | minimum temperature of May | °C |
| Tmin6 | minimum temperature of ‌June | °C |
| Tmin7 | minimum temperature of ‌July‌ | °C |
| Tmin8 | minimum temperature of ‌August‌ | °C |
| Tmin9 | minimum temperature of ‌September‌ | °C |
| Tmin10 | minimum temperature of ‌October‌ | °C |
| Tmin11 | minimum temperature of ‌November‌ | °C |
| Tmin12 | minimum temperature of ‌December‌ | °C |
| Vapr1 | water vapor pressure of January | kPa |
| Vapr2 | water vapor pressure of February | kPa |
| Vapr3 | water vapor pressure of ‌March‌ | kPa |
| Vapr4 | water vapor pressure of ‌April‌ | kPa |
| Vapr5 | water vapor pressure of May | kPa |
| Vapr6 | water vapor pressure of ‌June | kPa |
| Vapr7 | water vapor pressure of ‌July‌ | kPa |
| Vapr8 | water vapor pressure of ‌August‌ | kPa |
| Vapr9 | water vapor pressure of ‌September‌ | kPa |
| Vapr10 | water vapor pressure of ‌October‌ | kPa |
| Vapr11 | water vapor pressure of ‌November‌ | kPa |
| Vapr12 | water vapor pressure of ‌December‌ | kPa |
| Wind1 | wind speed of January | m/s |
| Wind2 | wind speed of February | m/s |
| Wind3 | wind speed of ‌March‌ | m/s |
| Wind4 | wind speed of ‌April‌ | m/s |
| Wind5 | wind speed of May | m/s |
| Wind6 | wind speed of ‌June | m/s |
| Wind7 | wind speed of ‌July‌ | m/s |
| Wind8 | wind speed of ‌August‌ | m/s |
| Wind9 | wind speed of ‌September‌ | m/s |
| Wind10 | wind speed of ‌October‌ | m/s |
| Wind11 | wind speed of ‌November‌ | m/s |
| Wind12 | wind speed of ‌December‌ | m/s |

**Table S3.** Suitability distribution areas for three host species of *Talaromyces marneffei* in China.

| **Species** | **Low** | **Medium** | **High** | **Very high** | **[High + Very high]** |
| --- | --- | --- | --- | --- | --- |
| *Rhizomys pruinosus* | 781.2240 | 83.2830 | 66.3767 | 30.8819 | 97.2586 |
| *Rhizomys sinensis* | 792.8663 | 108.1458 | 46.7153 | 14.0382 | 60.7535 |
| *Rhizomys sumatrensis* | 956.1615 | 4.0885 | 1.1319 | 0.3837 | 1.5156 |

*Note*: Area of each suitability class for three host species was calculated and shown in table. Unit: 10,000 km^2^.

**Table S4.** Occurrence records of three *Talaromyces marneffei* hosts from GBIF database.

| **Species** | **GBIF occurrence record link** |
| --- | --- |
| *Rhizomys pruinosus* | https://doi.org/10.15468/dl.tqfkcs |
| *Rhizomys sinensis* | https://doi.org/10.15468/dl.wx9js5 |
| *Rhizomys sumatrensis* | https://doi.org/10.15468/dl.haz4rd |
